# Disease modifying biomaterials for modulating mechanical allodynia in a preclinical model of rheumatoid arthritis

**DOI:** 10.1002/btm2.70054

**Published:** 2025-07-31

**Authors:** Maksim Dolmat, Julia Borges Paes Lemes, Wade T. Johnson, Elizabeth L. Wilkinson, Tony L. Yaksh, Nunzio Bottini, Nisarg J. Shah

**Affiliations:** ^1^ Department of Chemical and Nano Engineering University of California San Diego La Jolla California USA; ^2^ Department of Anesthesiology University of California San Diego La Jolla California USA; ^3^ Kao Autoimmunity Institute and Division of Rheumatology Cedars‐Sinai Medical Center Los Angeles California USA

**Keywords:** autoimmunity, biomaterials, pain

## Abstract

Pain is a key symptom associated with rheumatoid arthritis (RA) and can persist even in the context of overall disease control by standard‐of‐care disease modifying anti‐rheumatic drugs (DMARDs). Analgesic agents and corticosteroids are often used to supplement DMARDs for pain relief but lack disease modifying properties, and their sustained use carries adverse risks. In this work, we characterized the progression of pain sensitivity in the SKG mouse model of RA and evaluated the potential therapeutic interventions. Male and female SKG mice, after systemic mannan injection, developed a mechanical pain phenotype and joint swelling, with a strong inverse correlation between clinical arthritis scores and pain thresholds. To test potential interventions for pain alleviation, we evaluated all‐trans retinoic acid (ATRA)‐loaded poly(lactic‐co‐glycolic acid) microparticles (ATRA‐PLGA MP) administered via intra‐articular injection, which we have previously demonstrated to be disease‐modifying. The pain and inflammation patterns assessed by the von Frey test and clinical scoring showed ATRA‐PLGA MP monotherapy reduced inflammation and alleviated mechanical allodynia in arthritic SKG mice, an effect that was amplified by combination treatments with standard‐of‐care agents. In early‐stage arthritis, co‐administration with cytotoxic T‐lymphocyte‐associated protein (CTLA)‐4‐Ig, clinically known as abatacept, delayed disease progression and sustained the reduction of mechanical allodynia. In established arthritis, sequential treatment with the corticosteroid dexamethasone (Dex) reduced cumulative disease burden and reduced mechanical allodynia. These findings highlight the potential of combining ATRA‐PLGA MP with standard‐of‐care treatments as a potential strategy to enhance the efficacy and durability of disease modification and pain alleviation for arthritis management.

**Translational Impact Statement**

Pain is the biggest complaint of rheumatoid arthritis (RA) patients that often persists despite disease control with standard therapies. Using the SKG mouse model of RA, we characterized pain progression and evaluated all‐trans retinoic acid‐loaded microparticles as a localized intra‐articular therapy in combination with cytotoxic T‐lymphocyte‐associated protein‐4 Ig, a front‐line disease‐modifying agent, and dexamethasone, a corticosteroid used in pain management. We show combination treatment enhances and prolongs alleviation of arthritis severity and mechanical allodynia, supporting the potential of this approach in improving arthritis pain management and disease control.

## 1 INTRODUCTION

Joint pain is the most common symptom of inflammatory autoimmune arthropathies such as rheumatoid arthritis (RA).^1^, ^2^, ^3^ Even though disease‐modifying anti‐rheumatic drugs (DMARDs) have greatly improved treatment, pain can persist despite overall disease control, often with a poor prognosis.^4^, ^5^ On the other hand, improved pain outcomes have been associated with early treatment that sustains suppression of inflammation.^6^, ^7^, ^8^ However, such treatments rely on analgesic agents and/or steroids that are administered in addition to DMARDs, a strategy that increases iatrogenic risks of adverse events, including severe infections and cancer.^9^, ^10^, ^11^ There remains an unmet need for an anti‐arthritic immunoregulatory agent that operates as an adjunct to the standard‐of‐care treatment for addressing pain.

Pain assessments in rodent models of arthritis, including CIA, AIA, CAIA, and K/BxN, have revealed RA pain phenotypes that, in many respects, parallel those observed in humans.^12^, ^13^, ^14^ However, unlike the aforementioned arthritis models which require a specific antigen or antibody stimulus, BALB/c SKG mice develop spontaneous arthritis, which is accelerated with the injection of fungal components such as mannan, and recapitulates key pathophysiological features of human disease.^15^, ^16^ SKG mice carry a mutation in the ZAP‐70 gene, which impairs T cell receptor signaling and promotes the expansion of autoreactive T cells. SKG mice develop polyarthritis characterized by symmetric involvement of joints, and cartilage and bone erosions. The clinical profile has a chronic inflammatory phase that reliably mimics RA progression. While well‐established in the RA field, the SKG mice have not been characterized as an experimental model of inflammatory pain.

Here, we sought to characterize the pain phenotype in the SKG mouse model of autoimmune arthritis and test the potential of disease‐modifying agents for arthritis pain modulation at early and established stages. We characterized the pain behavior profiles of male and female arthritic SKG mice by measuring the mechanical and thermal hind paw thresholds and demonstrated a correlation of arthritis clinical scores and mechanical allodynia, with female mice potentially showing reduced mechanical hypersensitivity to arthritis pain. In addition, we tested the potential of a previously developed disease‐modifying agent, comprising biodegradable poly‐lactic‐co‐glycolic acid (PLGA) polymer microparticles (ATRA‐PLGA MP), by intra‐articular delivery in SKG mice on promoting analgesic effects.^17^ In agreement with our previous report, ATRA‐PLGA MP demonstrated a sustained immunomodulatory effect and significant disease attenuation. In combination with the cytotoxic T‐lymphocyte‐associated protein 4 immunoglobulin G (CTLA‐4‐Ig) DMARD in early‐stage arthritis, ATRA‐PLGA MP controlled disease and modified pain. When combined with Dex in established arthritis, ATRA‐PLGA MP improved disease control and modified pain, potentially minimizing the need for repeated corticosteroid administration. Together, these findings highlight the potential of ATRA‐PLGA MP as a complementary agent to existing arthritis treatments for sustained disease modulation together with pain and inflammation management.

## 2 RESULTS

### 2.1 Temporal profiling of pain behavior in SKG mice following mannan‐induced arthritis

**FIGURE 1:**
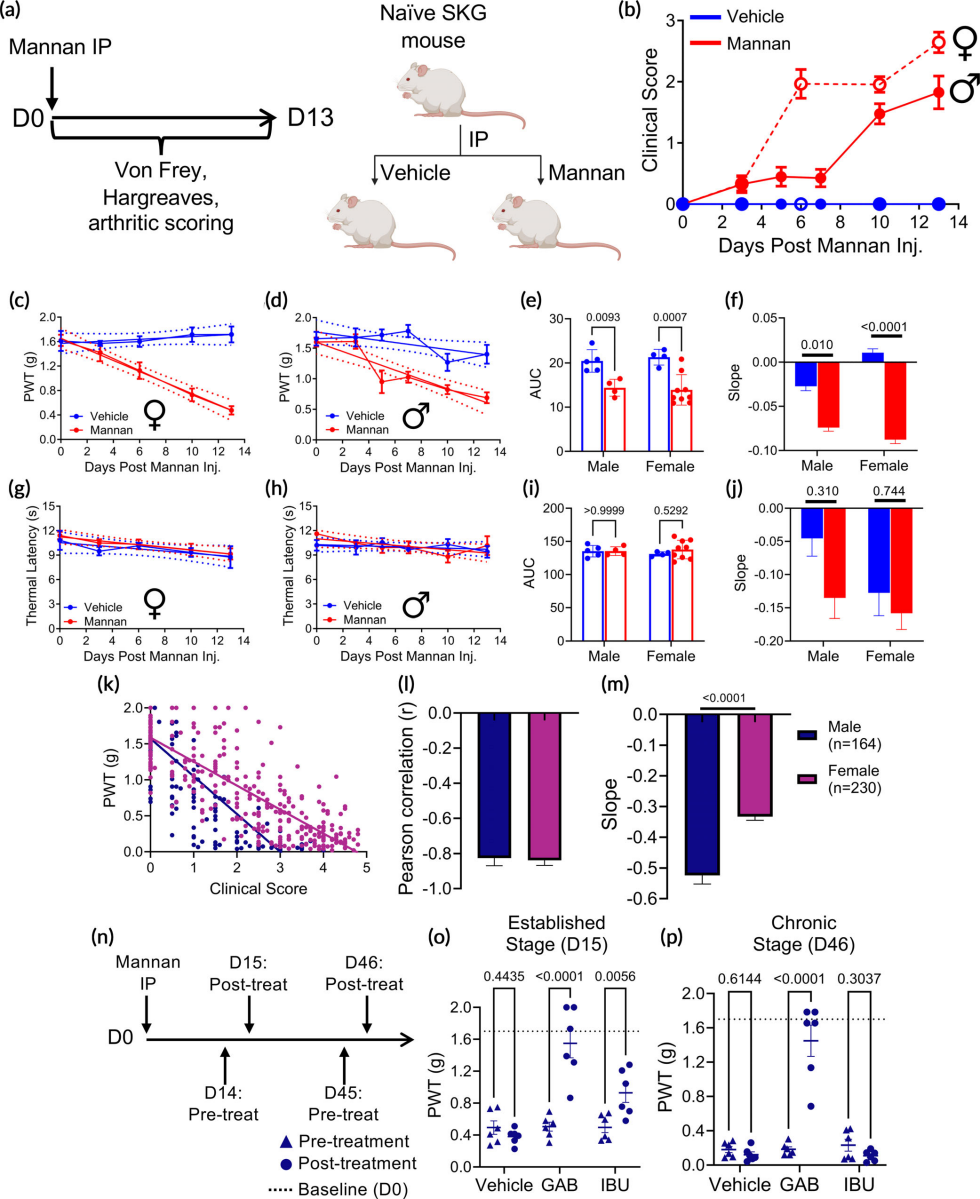
Temporal profiling of pain behavior in SKG mice following mannan‐induced arthritis. (a) Experimental design: SKG mice received an intraperitoneal (IP) injection of mannan or vehicle (1 × PBS) on day 0 to induce arthritis. Disease progression was monitored through clinical scoring, and pain behavior was assessed via von Frey and thermal paw withdrawal latency tests through day 13. (b) Clinical arthritis scores were significantly elevated in mannan‐treated mice compared to vehicle controls in both sexes, indicating progressive arthritis development. Mechanical allodynia, assessed via von Frey testing in female (c) and male (d) mice, showed reduced paw withdrawal thresholds following mannan administration. Quantification of mechanical allodynia: AUC (e) and slope analysis (f) confirmed significant mechanical hypersensitivity in mannan‐treated mice. Thermal hyperalgesia, measured via thermal withdrawal latency, did not significantly differ between mannan‐ and vehicle‐treated groups in female (g) or male (h) mice. Quantification of thermal sensitivity: AUC (i) and slope analysis (j) showed no significant differences between mannan and vehicle groups, indicating a lack of thermal hyperalgesia development. (k) Correlation between mechanical allodynia and arthritis severity: A scatter plot of paw withdrawal thresholds (PWT) and clinical arthritis scores (day 0–day 13) in male (dark blue) and female (purple) SKG mice revealed a significant inverse correlation in both sexes. Quantification of pain‐clinical score correlation: Pearson correlation coefficients (l) and slope analysis (m) confirmed a strong negative correlation between arthritis severity and mechanical allodynia, with each data point in (k) representing a unique paired clinical score vs. PWT pooled from experiments represented in Figures 1 and 4 collected throughout the course of the study. (n) Study design for evaluating arthritis pain response at established (day 15) and chronic (day 46) stages: SKG mice received an IP injection of mannan on day 0, with pre‐treatment pain assessments on days 14 and 45. On days 15 and 46, mice were treated with gabapentin (GAB, 100 mg × kg^−1^) or ibuprofen (IBU, 100 mg × kg^−1^), followed by post‐treatment mechanical allodynia assessments. At established stage (day 15), gabapentin and ibuprofen significantly improved mechanical allodynia one‐hour post‐treatment compared to vehicle (o). At chronic stage (day 46), gabapentin continued to alleviate mechanical hypersensitivity, whereas ibuprofen had no significant effect (p). Data are presented as mean ± SEM (b–d, f, g–h, j, l, m, o, p), mean ± SD (e), (i) with *n* > 4 per group. Statistical significance was determined by two‐way ANOVA (e, i, o, p) or F‐test for the slopes analysis (f, j, m). For the AUC quantification, the baseline was set to 0.

Following intraperitoneal (IP) injection of mannan or vehicle (1 × PBS) on day 0, female and male SKG mice were evaluated for arthritis progression and mechanical allodynia as a measure of pain through day 13 (Figure 1a). Arthritis was assessed through aggregated clinical scores of all paws (Figure 1b), mechanical allodynia was evaluated using von Frey mechanical sensitivity testing (Figure 1c–f), and thermal hyperalgesia was assessed through thermal paw withdrawal latency (Figure 1g–j). Responses from the left and right hind paws were analyzed and found to be similar in both sexes (Figure S1) and the average from both hind paws was used for further analysis.

Both female and male SKG mice developed arthritis following mannan injection. Control mice, which received 1 × PBS, remained free of visible arthritis (Figure 1b). Clinical scores indicated that arthritis onset occurred earlier in females, with signs of inflammation appearing by day 3, reaching a clinical score of 2.0 ± 0.2 by day 6, and peaking at 2.6 ± 0.2 by day 13. In males, arthritis first appeared on day 5 with a clinical score of 0.5 ± 0.2, increasing to 1.8 ± 0.3 by day 13. The progression of arthritis severity followed a similar trend in both sexes, though female mice exhibited greater disease severity, consistent with previous observations of sex differences in arthritis models.^18^, ^19^

Mannan‐induced arthritis resulted in a significant reduction in mechanical paw withdrawal threshold (PWT) compared to baseline and control groups in both female and male mice (Figure 1c, d). In females, PWT decreased from 1.62 ± 0.01 g at baseline to 1.13 ± 0.14 g by day 6, and further declined to 0.48 ± 0.07 g by day 13, representing a nearly fourfold reduction. Similarly, males exhibited a significant reduction in PWT, from 1.60 ± 0.07 g at baseline to 0.95 ± 0.19 g by day 5, reaching 0.69 ± 0.09 g by day 13. AUC analysis confirmed decreased mechanical threshold in both sexes. The AUC for female mice was 14 ± 3 compared to 21 ± 2 in controls, and for male mice, it was 14 ± 2 compared to 21 ± 3 in controls (Figure 1e). Linear regression analysis further supported declines in PWT in mannan‐injected mice, with slopes of −0.088 ± 0.010 in females and −0.074 ± 0.010 in males, in contrast to a modest change in vehicle‐injected mice (Figure 1f).

Thermal hyperalgesia was assessed using the Hargreaves method by measuring thermal paw withdrawal latency.^20^, ^21^ Unlike the pronounced effects observed in mechanical allodynia, thermal sensitivity remained unchanged in both sexes throughout the study (Figure 1g, h). AUC and linear regression analyses showed no significant differences between mannan‐ and vehicle‐treated groups, indicating that mannan‐induced arthritis primarily affects PWT rather than thermal paw withdrawal latency (Figure 1i, j). These findings are consistent with previous studies in K/BxN and late‐stage CAIA models, where mechanical hypersensitivity was demonstrated to be the predominant pain modality.^22^, ^23^, ^24^ Based on these results, further thermal sensitivity analysis was not conducted.

To characterize the relationship between PWT and arthritis progression in SKG mice, we correlated clinical scores and PWT in both male and female mice analyzed data from day 0 to day 13 (Figure 1k). Data were pooled from experiments in Figures 1 and 4, as both studies included cohorts in which arthritis developed without intervention. Pearson correlation analysis revealed a strong inverse relationship between clinical scores and mechanical thresholds in both sexes (*r* < −0.8), supporting that increased arthritis severity correlated with heightened pain sensitivity (Figure 1l). Linear regression analysis further quantified the relationship, revealing a significant difference in the slopes of the regression lines between males and females (Figure 1m). Specifically, the correlation for male mice was associated with a slope of −0.52 ± 0.03, greater than that of female mice (−0.33 ± 0.01), suggesting that while inflammation‐driven mechanical allodynia is evident in both male and female SKG mice, female SKG mice show lower mechanical allodynia during arthritis.

To investigate the effect of standard pain interventions on mechanical allodynia in SKG mice, the PWT was evaluated following treatment with gabapentin (GAB, 100 mg × kg^−1^, IP), ibuprofen (IBU, 100 mg × kg^−1^, IP), or vehicle (10% DMSO/10% Tween 80 in PBS, IP) at both established (day 15 post‐mannan) and chronic (day 46) stages of arthritis (Figure 1n). On day 14, pre‐treatment PWT was measured prior to the administration of treatments on day 15. A 1‐h post‐treatment assessment showed that both GAB and IBU significantly reduced mechanical allodynia, as indicated by increased PWT relative to their respective pre‐treatment baselines, whereas no significant change was observed in the vehicle‐treated group (*p* < 0.05) (Figure 1o). Specifically, the PWT of the GAB‐treated group increased from 0.51 ± 0.06 g to 1.55 ± 0.18 g, and the IBU‐treated group shifted from 0.50 ± 0.07 g to 0.93 ± 0.11 g, pre‐ and 1‐hour post‐treatment, respectively, while the PWT remained unchanged in the vehicle group. The analyses were repeated in chronic arthritis (day 46 post‐mannan), with pre‐treatment mechanical threshold assessments conducted on day 45, and treatment on day 46. In contrast to the established stage assessments, where both GAB and IBU showed improvements, PWT measured 1‐h post‐treatment showed significant improvement only in the GAB‐treated group, with recovery from 0.18 ± 0.03 g pre‐treatment to 1.45 ± 0.18 g post‐treatment. No changes were seen in the IBU (0.23 ± 0.07 g pre‐treatment to 0.11 ± 0.02 g post‐treatment) and vehicle (0.18 ± 0.04 g pre‐treatment to 0.12 ± 0.03 g post‐treatment) groups (Figure 1p). These results are comparable to the results reported in the KBxN model and suggest a change in the contribution of cyclooxygenase‐mediated sensitization and suggest a neuropathic‐like phenotype.^25^ Neither ibuprofen nor gabapentin significantly altered the clinical score 1‐h post‐treatment at the chronic disease stage, supporting that gabapentin intervention modulates pain without affecting inflammation (Figure S2).

### 2.2 Optimizing intra‐articular delivery of all‐trans retinoic acid using PLGA microparticles

**FIGURE 2:**
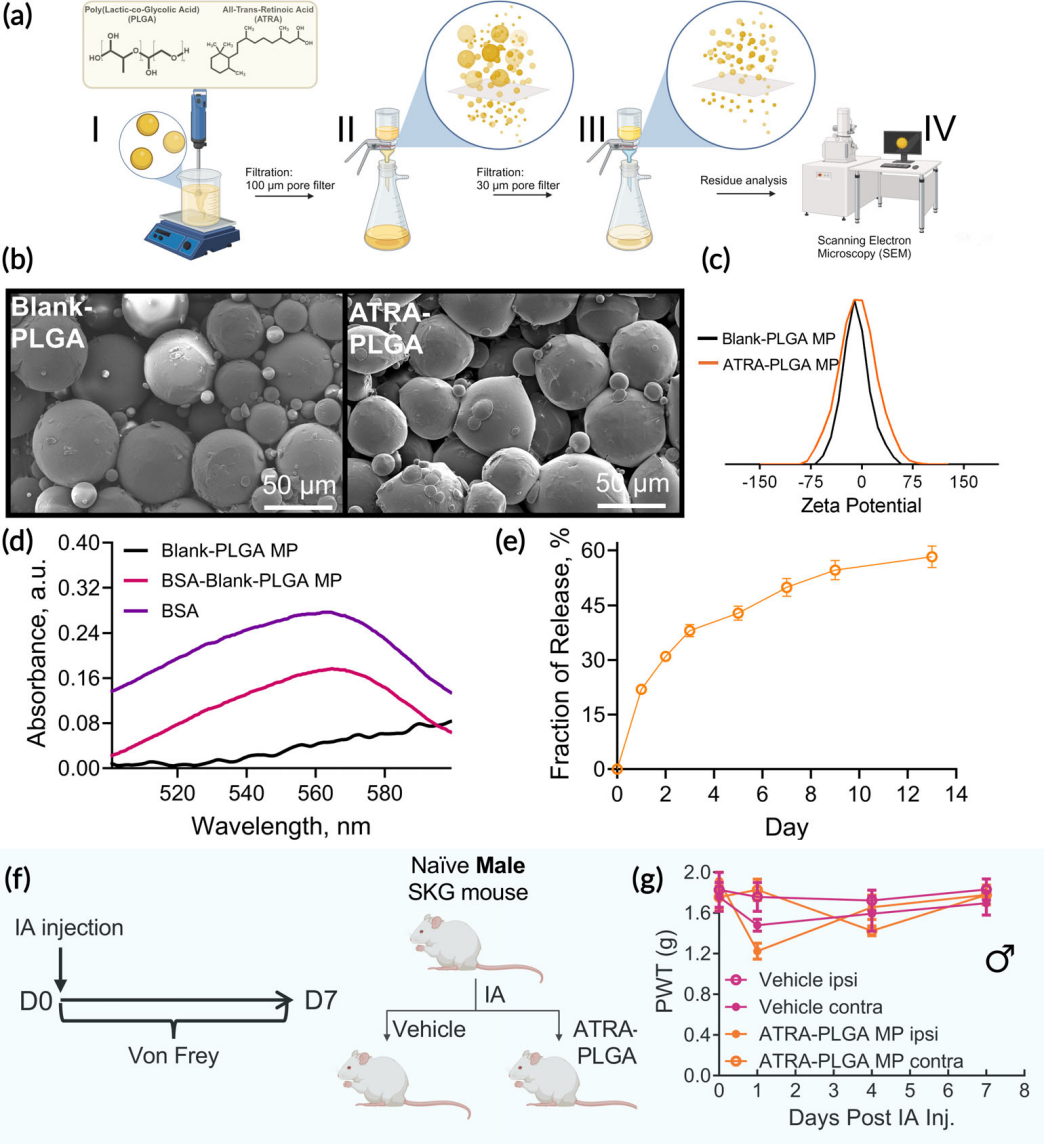
Synthesis and characterization of ATRA‐loaded PLGA macroparticles (ATRA‐PLGA MP). (a) Schematic representation of macroparticle (MP) synthesis: Empty (Blank‐PLGA) and ATRA‐loaded (ATRA‐PLGA) MP were synthesized via an oil‐in‐water emulsion method (I), followed by sequential filtration using 100 μm (II) and 30 μm (III) pore filters to remove agglomerates and small particles. Residual particles from the final filtration step were analyzed using scanning electron microscopy (SEM) (IV). (b) SEM images of Blank‐PLGA MP and ATRA‐PLGA MP confirm a spherical morphology with an average particle size of ~50 μm. (c) Zeta potential analysis reveals a near‐neutral surface charge for both Blank‐PLGA MP and ATRA‐PLGA MP. (d) Protein corona formation on Blank‐PLGA MP: UV–vis absorbance measurements indicate bovine serum albumin (BSA) adsorption on Blank‐PLGA MP, suggesting surface interactions. (e) In vitro ATRA release profile: ATRA‐PLGA MP exhibit an initial burst release phase within the first 3 days, followed by sustained release over 14 days in the presence of 10 mg × kg^−1^ BSA. The data are presented as the mean ± SD, with experiments conducted in triplicate. (f) Assessment of pain induction by ATRA‐PLGA MP in naïve SKG mice: Naïve male SKG mice received an intra‐articular (IA) injection of ATRA‐PLGA MP or vehicle (1 × PBS) in one ankle, followed by mechanical allodynia assessment via von Frey testing in the ipsilateral (ipsi) and contralateral (contra) paws on days 1, 4, and 7 post‐injection. (g) PWT indicates no significant pain induction following IA injection of ATRA‐PLGA MP compared to vehicle‐treated controls. Data are presented as mean ± SEM (*n* > 4).

We have previously demonstrated that all‐trans retinoic acid (ATRA) encapsulated in poly(lactic‐co‐glycolic acid) PLGA‐based microparticles (ATRA‐PLGA MP) promote sustained joint‐localized release, reducing inflammation by enhancing disease‐protective *T*_reg_, which is disease modifying in arthritic SKG mice.^17^ This prior observation set the stage for investigating how ATRA‐PLGA MP might modify pain in SKG mice. Empty (Blank‐PLGA MP) and ATRA‐loaded (ATRA‐PLGA MP) particles were synthesized using an oil‐in‐water emulsion method (Figure 2a, step I). The synthesis process included rigorous double filtration steps to ensure homogeneity: a 100 μm filter to remove large aggregates (Figure 2a, step II) and a 30 μm filter to eliminate small particles (Figure 2a, step III). Residual particles were washed four times with sterile deionized water before being analyzed via scanning electron microscopy (SEM) to confirm the success of the filtration process (Figure 2a, step IV).

SEM imaging showed spherical morphologies for both Blank‐PLGA MP and ATRA‐PLGA MP, with diameters averaging approximately 50 ± 8 μm (Figure 2b). ATRA loading was ~1.7%, comparable to our previous report.^17^ Zeta potential analysis showed a near‐neutral surface charge for both Blank‐PLGA MP and ATRA‐PLGA MP (Figure 2c). PLGA itself lacks substantial ionizable groups, resulting in a generally neutral net charge.^26^ Since ATRA is a weak acid with a pKa around 4.76,^27^ it remains predominantly in its neutral, non‐ionized form at physiological pH (7.4), and therefore, the incorporation of ATRA into PLGA MP does not affect the particle surface charge.

Prior work has shown that protein corona formation on particle surfaces can influence particle behavior,^28^, ^29^ we investigated the effect of changing the concentration of albumin, the most abundant protein in synovial fluid (~12 mg × mL^−1^),^30^ on Blank‐PLGA MP. Blank‐PLGA MP were incubated with a bovine serum albumin (BSA, 10 mg × mL^−1^) at 37°C for 2 h to allow protein adsorption. After incubation, the particles were centrifuged (1000 g for 5 min), washed three times via resuspension in 1 × PBS to remove any unbound protein, and analyzed using a bicinchoninic acid (BCA) assay to quantify protein binding. The maximum absorbance peak at 562 nm was unchanged for both BSA alone and Blank‐PLGA MP incubated in BSA (Figure 2d). Blank‐PLGA MP that were not exposed to BSA showed no absorbance in this region, thus confirming BSA adsorption on the surface of Blank‐PLGA MP. This study was not conducted with ATRA‐PLGA MP due to interactions between ATRA and BCA assay reagents.

In vitro ATRA release studies were conducted using release media containing 10 mg × mL^−1^ BSA to simulate synovial fluid conditions (Figure 2e). To further investigate the impact of protein corona formation on ATRA release kinetics, additional studies were performed under low (0.1 mg × mL^−1^) and high (50 mg × mL^−1^) BSA concentrations. The high concentration of BSA was selected to reflect the albumin levels typically found in serum, making it relevant for the potential intravenous administration of the particles.^31^ ATRA release at a BSA concentration of 10 mg × mL^−1^ showed a biphasic pattern, with an initial burst phase over the first 3 days likely due to surface‐bound ATRA, transitioning to a slower, sustained release governed by polymer degradation and diffusion, supporting prolonged drug delivery (Figure 2e). The release profiles were consistent across various BSA concentrations, with an initial burst phase during the first 3 days followed by a more gradual and sustained release; however, the release rate and overall ATRA release fraction were influenced by the concentration of BSA (Figure S3a). A higher BSA concentration of 50 mg × mL^−1^ led to a more pronounced burst release, with a slope of 19.8 ± 2.2 during the initial 3 days, compared to 14.1 ± 1.8 at 10 mg × mL^−1^. Reducing the BSA concentration to 0.1 mg × mL^−1^ further decreased the burst release by half to 6.1 ± 0.2 (Figure S3b). Increasing the BSA concentration from 0.1 mg × mL^−1^ to 10 mg × mL^−1^ doubled the release from PLGA microparticles within the first 3 days, while raising it to 50 mg × mL^−1^ tripled the release compared to 0.1 mg × mL^−1^. Over 13 days, increasing BSA from 0.1 to 10 mg × mL^−1^ elevated the cumulative ATRA release fraction from ~35% to ~50%, with no further increase at 50 mg × mL^−1^. To more closely mimic the complexity of synovial fluid, ATRA release was evaluated in an artificial synovial fluid mimic with physiologically relevant viscosity (500 cP) and 1.5 mg × mL^−1^ BSA, hyaluronic acid, mucoprotein, and urea, among other components. ATRA release was significantly slower during the initial 3 days. The cumulative release of ATRA over 13 days in artificial synovial fluid was ~7% (Figure S3c).

To evaluate if intra‐articular injections were associated with mechanical allodynia, naïve SKG mice were administered IA injections of either vehicle (1 × PBS) or ATRA‐PLGA MP. PWT were assessed on days 1, 4, and 7 post‐injection and measurements compared between the ipsilateral and contralateral hind paws (Figure 2f). On day 1 following injection, a reduction in PWT in the injected paw was measured in ATRA‐PLGA MP mice. However, this effect was transient, and no significant differences between the ipsilateral and contralateral paws were observed on days 4 and 7, indicating a resolution of injection‐induced mechanical allodynia.

### 2.3 Evaluating pain modification in early‐stage arthritis

DMARDs are the standard‐of‐care for treating arthritis. Examples include JAK inhibitors like tofacitinib (Tofa)^32^, ^33^ and biologics such as tumor necrosis factor inhibitors (TNF) or cytotoxic T‐lymphocyte‐associated protein (CTLA‐4‐Ig)^34^ being among the most common strategies.

Given the widespread use of JAK inhibitors in RA treatment,^35^ we initially tested the efficacy of Tofa in SKG mice at a dose of 15 mg × mL^−1^ from day 3 onward but observed no significant therapeutic benefit (Figure S4). Arthritis studies in which tofacitinib has demonstrated efficacy have used a delayed‐onset arthritis model (e.g., Zymosan A) with treatment initiated at the onset of disease, often delivered continuously (e.g., via Alzet mini‐pumps) or with higher‐frequency dosing regimens (10 mg/kg, BID).^33^ In contrast, in our studies, arthritis was induced using mannan, which produces a rapid‐onset inflammatory response with visible clinical signs by day 3. Tofacitinib was administered via a single oral dose of 15 mg/kg starting on day 3, when signs of arthritis were already present. The differences in disease model, dosing strategy, and route of administration in our study likely contributed to the lack of efficacy.

**FIGURE 3:**
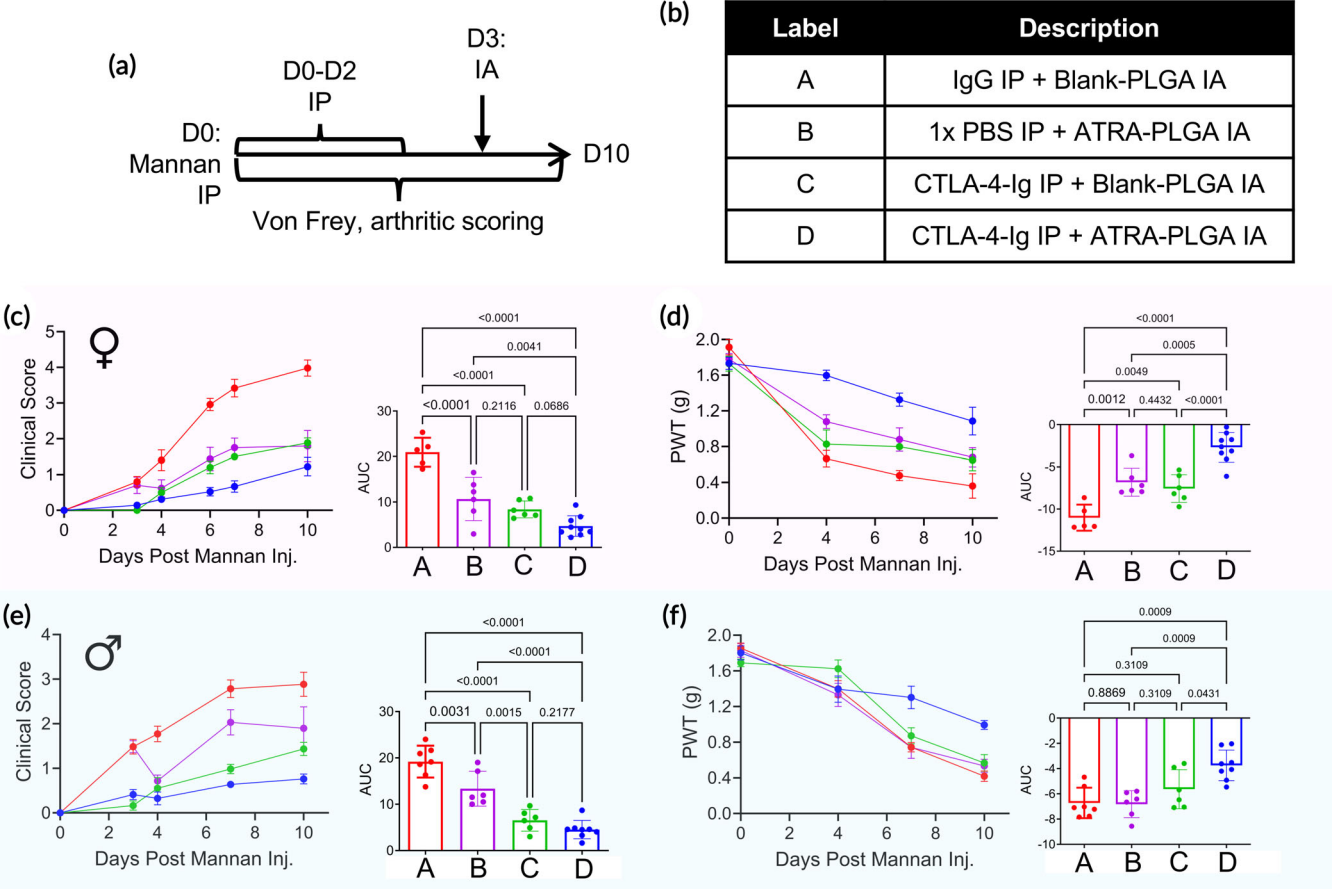
Therapeutic evaluation of ATRA‐PLGA MP in early‐stage arthritis in SKG mice. (a) Study design: Arthritis was induced in SKG mice via IP injection of mannan on day 0. Mice received daily IP injections of CTLA‐4‐Ig (10 mg × kg^−1^), IgG (10 mg × kg^−1^), or vehicle (1 x PBS) from day 0 to day 2. On day 3, mice were administered IA either Blank‐PLGA MP (control) or ATRA‐PLGA MP. (b) Mice were divided into four experimental treatment groups. Clinical arthritis scores in female (c) and male (e) mice, including an inset showing the AUC analysis of scores throughout the study. Mechanical allodynia in female (d) and male (f) mice, assessed via von Frey testing with corresponding AUC analysis. Clinical score and mechanical allodynia data are shown as mean ± SEM (*n* > 5), while AUC data are presented as mean ± SD. For panels (c) and (e), the baseline for AUC was set to 0, while for panels (d) and (f), the baseline was defined as the group average on day 0. Statistical analysis was performed using one‐way ANOVA.

CTLA‐4‐Ig, a widely used biologic DMARD that operates by competitively inhibiting the CD28‐CD80/86 co‐stimulatory pathway, has been shown to have potential for preventing arthritis in clinical trials.^34^, ^36^ Our previous work also identified increased CTLA‐4 expression in joint‐localized dendritic cells and CD4^+^ T cells following treatment with calcitriol‐loaded nanoparticles, which was associated with reduced arthritis severity and protection against bone and cartilage damage.^37^ Based on these findings, we evaluated the efficacy of ATRA‐PLGA MP at the early stage of arthritis, benchmarking it against CTLA‐4‐Ig and assessing the potential for added therapeutic benefit with combination therapy. CTLA‐4‐Ig was administered daily via IP injection from day 0 to day 2 at a dose of 10 mg × kg^−1^, while a similarly dosed IgG group served as a negative control. To evaluate the effects of ATRA‐PLGA MP alone, a separate group received a vehicle (1 x PBS) via IP injection from day 0 to day 2, followed by a single IA injection of ATRA‐PLGA MP on day 3. A combination therapy group received CTLA‐4‐Ig from day 0 to day 2, followed by a single IA injection of ATRA‐PLGA MP on day 3 (Figure 3a, b).

Clinical scores in female SKG mice were compared between treatment groups (Figure 3c). Control IgG treatment had no effect on arthritis severity. ATRA‐PLGA MP treatment alone comparatively slowed disease progression, with clinical scores of 0.6 ± 0.2 on day 4 and 1.8 ± 0.4 on day 10. CTLA‐4‐Ig monotherapy effectively delayed the onset of arthritis during the treatment period through day 3. However, arthritis developed once CTLA‐4‐Ig administration ceased, with clinical scores of 1.9 ± 0.1 by day 10. The combination of CTLA‐4‐Ig and ATRA‐PLGA MP treatment improved arthritis control, with lower average clinical scores than other groups throughout the experiment. Minimal signs of arthritis were observed (clinical score of 0.3 ± 0.1) on day 4, and subsequently increased slowly to 0.7 ± 0.2 on day 7, and 1.2 ± 0.3 by day 10. AUC analysis showed that combination therapy was significantly better than IgG and ATRA‐PLGA MP treatment alone, but comparable to CTLA‐4‐Ig treatment (Figure 3c).

Mechanical allodynia was assessed through PWT measurements on both hind paws. No significant differences were observed between ipsilateral and contralateral paws (Figure S5a). The PWT in IgG‐treated mice progressively decreased from 1.91 ± 0.09 g on day 0 to 0.66 ± 0.09 g by day 4 and 0.36 ± 0.14 g by day 10 (Figure 3d). Comparatively, ATRA‐PLGA MP treated‐mice alleviated mechanical allodynia decline as assessed by a higher PWT of 1.78 ± 0.06 g on day 0, 1.08 ± 0.08 g on day 4, and 0.68 ± 0.11 g on day 10. CTLA‐4‐Ig monotherapy delayed the onset of mechanical allodynia during the treatment phase; however, the PWT began to decrease after treatment, following a similar trajectory to the ATRA‐PLGA MP group, changing from 1.73 ± 0.08 g on day 0 to 0.83 ± 0.16 g on day 4 and 0.65 ± 0.12 g on day 10. In contrast, the combination therapy group significantly sustained pain alleviation compared to both ATRA‐PLGA MP and CTLA‐4‐Ig monotherapy. PWT remained near baseline through day 4 (1.73 ± 0.07 g on day 0 to 1.60 ± 0.06 g on day 4). Reductions in PWT by day 7 (1.33 ± 0.07 g) and day 10 (1.09 ± 0.15 g) were significantly less than in other groups. These findings suggest that the addition of ATRA‐PLGA MP to CTLA‐4‐Ig therapy might not only delay the progression of arthritis but also provide more effective and prolonged control of the pain. AUC analysis showed that combination therapy significantly improved mechanical allodynia compared to other groups, with AUC values of −11 ± 2 for the IgG‐treated group, −7 ± 2 for the ATRA‐PLGA MP group, −8 ± 2 for the CTLA4‐Ig group, and −3 ± 2 for the combination therapy group (Figure 3d).

To extend the characterization to male SKG mice, the study was repeated. A trend similar to that in female mice was observed between treatment groups (Figure 3e, f). The onset of arthritis in control IgG‐treated male mice was more gradual compared to female mice. Clinical signs were observed on day 3 and progressed steadily to scores of 1.8 ± 0.2 on day 4, 2.8 ± 0.2 on day 7, and 2.9 ± 0.3 by day 10. Monotherapy treatment with ATRA‐PLGA MP significantly reduced arthritis progression, reducing clinical scores from 1.5 ± 0.1 on day 3 (prior to IA injection) to 0.7 ± 0.1 on day 4 post‐treatment. ATRA‐PLGA MP treatment maintained low clinical scores of 2.0 ± 0.3 on day 7 and 1.9 ± 0.5 on day 10, significantly lower than those observed in the IgG‐treated group (Figure 3e). CTLA‐4‐Ig monotherapy provided strong initial protection from arthritis, maintaining low clinical scores through day 3. However, arthritis symptoms emerged after CTLA‐4‐Ig treatment, with scores increasing from 0.6 ± 0.2 on day 4 to 1.0 ± 0.1 on day 7 and 1.4 ± 0.2 on day 10. Combination ATRA‐PLGA MP and CTLA‐4‐Ig treatment slowed disease progression compared with CTLA‐4‐Ig monotherapy alone, as reflected by clinical scores of 0.3 ± 0.1 on day 4, 0.6 ± 0.1 on day 7, and 0.8 ± 0.1 on day 10. AUC analysis showed that combination therapy was significantly better than IgG and ATRA‐PLGA MP treatment alone, but comparable to CTLA‐4‐Ig treatment (Figure 3e).

Mechanical allodynia was assessed through PWT measurements on both hind paws. As in female mice, no significant differences were observed between ipsilateral and contralateral paws (Figure S5b). PWT in IgG‐treated mice reduced from 1.86 ± 0.06 g at baseline (day 0) to 1.40 ± 0.09 g on day 4, 0.74 ± 0.05 g on day 7, and 0.42 ± 0.06 g on day 10 (Figure 3f). Despite its effect on modulating clinical arthritis, ATRA‐PLGA MP did not prevent the decline in PWT, which was similar to the IgG group. Interestingly, CTLA‐4‐Ig monotherapy and combination therapy exhibited a similar trend to the control IgG group by day 4, despite providing strong protection against clinical arthritis symptoms. However, while the PWT in CTLA‐4‐Ig‐treated mice continued to decrease, combination therapy effectively stabilized and slowed the progression of mechanical allodynia, maintaining higher PWTs of 1.30 ± 0.13 g on day 7 and 1.00 ± 0.05 g on day 10. AUC analysis further validated the enhanced efficacy of combination therapy over the individual monotherapies, showing AUC values of −7 ± 1 for the IgG‐treated group, −7 ± 1 for the ATRA‐PLGA MP group, −6 ± 2 for the CTLA‐4‐Ig group, and −4 ± 1 for the combination therapy group (Figure 3f). These findings collectively suggest that while ATRA‐PLGA MP and CTLA‐4‐Ig monotherapies provide protection against arthritis progression, they do not prevent the development of mechanical allodynia. In contrast, combination therapy improved protection against the development of mechanical allodynia.

### 2.4 Evaluating pain modification in the context of corticosteroid treatment

**FIGURE 4:**
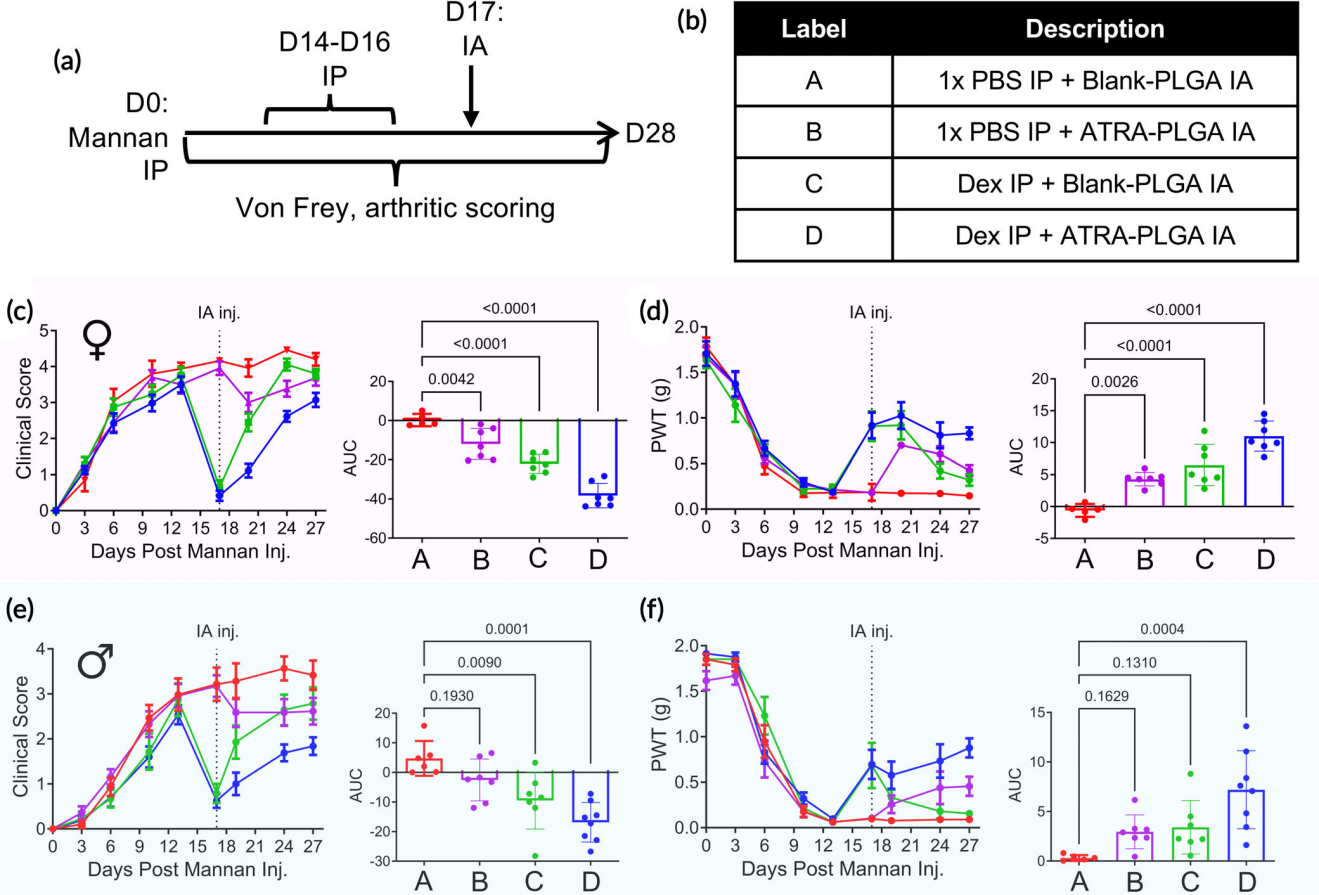
Assessing pain alleviating potential of ATRA‐PLGA MP in SKG mice at established stage of arthritis. (a) Study design: Arthritis was induced in SKG mice via IP injection of mannan on day 0. Dexamethasone (Dex; 5 mg × kg^−1^ for females, 1 mg × kg^−1^ for males) was administered daily via IP injection from days 14 to 16. On day 17, mice received an IA injection of ATRA‐PLGA MP. Clinical arthritis scores and mechanical allodynia were assessed until the study endpoint on day 28. (b) Mice were divided into four experimental treatment groups. Clinical arthritis scores in female (c) and male (e) mice, with insets displaying AUC analysis across the groups. von Frey testing of mechanical allodynia in female (d) and male (f) mice, accompanied by AUC analysis comparing the groups. For panels (c)–(f), the baseline was set to the group average on day 14. Clinical score and mechanical allodynia data are shown as mean ± SEM (*n* > 5), while AUC data are presented as mean ± SD. Statistical significance was determined using one‐way ANOVA.

Corticosteroids such as dexamethasone (Dex) are sometimes used for pain and inflammation management; however, the treatment effect is not durable or disease modifying.^38^, ^39^ To potentially augment the effect of steroids, we evaluated the therapeutic efficacy of ATRA‐PLGA MP in preventing arthritis progression and the associated pain after Dex. SKG mice with mannan‐induced arthritis received daily IP injections of Dex or vehicle (4.75 w/v% ethanol in PBS) from days 14 to 16, followed by IA administration of either ATRA‐PLGA MP or Blank‐PLGA MP (control) on day 17 (Figure 4a). Four treatment groups were compared: a group receiving vehicle IP and Blank‐PLGA IA, an ATRA‐PLGA MP monotherapy group, a Dex monotherapy group, and a combination therapy group receiving both IP Dex and IA ATRA‐PLGA MP (Figure 4b).

The clinical scores for all groups followed a similar trajectory until day 13, with an average score of approximately 3.7 in female mice by day 13 (Figure 4c). At this stage, inflammation in SKG mice typically reaches a peak, as observed in the control group (IP vehicle + Blank‐PLGA MP IA), where clinical scores remained relatively stable, at 4.2 ± 0.1 on day 17 and 4.2 ± 0.2 on day 27. ATRA‐PLGA MP monotherapy significantly reduced clinical scores following IA administration on day 17, dropping from 4.0 ± 0.2 pre‐treatment to 3.0 ± 0.3 by day 20. Subsequently, clinical scores remained low at 3.4 ± 0.2 on day 24 and 3.7 ± 0.2 on day 27.

To compare these effects with corticosteroid treatment, mice were administered Dex via IP injections on days 14–16 to potently suppress arthritis. Initial studies using 1 mg × kg^−1^ Dex in female mice resulted in only partial arthritis suppression (Figure S6). However, increasing the dose to 5 mg × kg^−1^ strongly suppressed arthritis, with clinical scores averaging 0.6 ± 0.2 on day 17. After Dex administration, arthritis rapidly re‐established in the absence of an additional therapy, with an increase in clinical scores to 2.4 ± 0.3 on day 20 and 3.8 ± 0.1 on day 27, which was comparable to the control (vehicle IP, Blank‐PLGA MP IA) group. This observation confirmed the transient nature of Dex monotherapy in SKG mice. In contrast, the combination of Dex with ATRA‐PLGA MP significantly reduced cumulative disease burden, with clinical scores of 1.1 ± 0.2 on day 20 and 3.1 ± 0.2 on day 27, significantly lower than either monotherapy alone. AUC analysis confirmed that combinational therapy improved arthritis control compared to individual treatments, with AUC values of 0 ± 3 for the control group, −12 ± 8 for the ATRA‐PLGA MP group, −22 ± 5 for the Dex group, and −38 ± 6 for the combination therapy group (Figure 4c).

To evaluate the analgesic effects of ATRA‐PLGA MP alone and in combination with Dex, mechanical allodynia was assessed using PWT assessments (Figure 4d). All groups showed declining PWT with arthritis progression, from approximately ~1.7 g on day 0 to ~0.2 g on day 13. The control group (vehicle IP + Blank‐PLGA IA) showed no improvement in mechanical allodynia, with PWT values remaining at 0.18 ± 0.03 g on day 20 and 0.15 ± 0.02 g on day 27. ATRA‐PLGA MP monotherapy significantly alleviated mechanical allodynia, increasing PWT from 0.18 ± 0.03 g on day 17 to 0.70 ± 0.03 g on day 20, 0.60 ± 0.09 g on day 24, and slight decrease by day 27 to 0.43 ± 0.06 g. The transient nature of Dex monotherapy in arthritis suppression was also reflected in mechanical allodynia. While Dex‐treated mice experienced an initial recovery in PWT to 0.90 ± 0.16 g on day 17, mechanical allodynia returned as arthritis symptoms reappeared, with PWT progressively declining to 0.42 ± 0.08 g on day 24 and 0.32 ± 0.06 g on day 27. However, the combination of Dex and ATRA‐PLGA MP significantly alleviated mechanical allodynia, with PWT improving from 0.92 ± 0.14 g on day 17 to 1.02 ± 0.15 g on day 20 and 0.83 ± 0.07 g on day 27. AUC analysis further confirmed the enhanced analgesic effect of the combination therapy compared to monotherapies, with AUC values of −1 ± 1 for the control group, 4 ± 1 for the ATRA‐PLGA MP group, 7 ± 3 for the Dex group, and 11 ± 2 for the combination therapy group (Figure 4d).

To investigate the effect in male mice in established arthritis, the study was repeated (Figure 4e, f). Following mannan induction, male mice developed arthritis with clinical scores peaking at approximately ~2.8 on day 13, which was lower in severity compared to female mice. In the control (vehicle IP, Blank‐PLGA MP IA) group, the clinical scores were 3.3 ± 0.4 on day 20 and 3.4 ± 0.3 on day 27. ATRA‐PLGA MP monotherapy reduced the clinical score from 3.2 ± 0.2 on day 17 to 2.6 ± 0.3 on day 20, with sustained suppression through day 27, where the score remained at 2.6 ± 0.3.

While female mice exhibited more severe arthritis requiring a higher Dex dose (5 mg × kg^−1^), male mice were more responsive to a lower dose (1 mg × kg^−1^), achieving a comparable therapeutic effect. Dex treatment reduced clinical scores from 2.8 ± 0.4 on day 14 to 0.8 ± 0.2 on day 17. However, consistent with observations in female mice, Dex monotherapy provided only transient arthritis suppression, with clinical scores rapidly increasing from 1.9 ± 0.4 on day 20 to 2.8 ± 0.4 by day 27. In contrast, combination therapy with Dex and ATRA‐PLGA MP reduced cumulative arthritis disease burden, with clinical scores of 1 ± 0.3 on day 20 and 1.8 ± 0.2 on day 27, which were significantly lower than either monotherapy alone. AUC analysis further confirmed the enhanced effect of the combination therapy compared to monotherapies, with AUC values of 5 ± 6 for the control group, −3 ± 7 for the ATRA‐PLGA MP group, −9 ± 10 for the Dex group, and −17 ± 7 for the combination therapy group (Figure 4e). Importantly, combination therapy appeared to be even more effective in male mice, likely due to the lower baseline disease severity, allowing for better arthritis control and reduced clinical signs.

As arthritis progressed, PWT declined from ~1.8 g on day 0 to ~0.07 g on day 13 (Figure 4f). The control group maintained low PWT values through day 27, with a final PWT of 0.09 ± 0.01 g. In contrast, ATRA‐PLGA MP monotherapy provided significantly alleviated mechanical allodynia, increasing PWT to 0.26 ± 0.10 g on day 20 and further to 0.46 ± 0.11 g on day 27. Dex monotherapy was associated with a transient analgesic effect, temporarily increasing PWT to 0.68 ± 0.24 g on day 17, but mechanical allodynia returned as arthritis re‐emerged, with PWT declining to 0.18 ± 0.08 g on day 24 and 0.16 ± 0.01 g on day 27. In contrast, combination therapy resulted in sustained alleviation of mechanical allodynia, with PWT increasing from 0.70 ± 0.16 g on day 17 to 0.58 ± 0.15 g on day 20 and remaining at 0.88 ± 0.11 g on day 27. AUC analysis further confirmed the enhanced analgesic effect of the combination therapy compared to monotherapies, with AUC values of 0.3 ± 0.3 for the control group, 3 ± 2 for the ATRA‐PLGA MP group, 3 ± 3 for the Dex group, and 7 ± 4 for the combination therapy group (Figure 4f).

Overall, results from both male and female SKG mice demonstrate that ATRA‐PLGA MP effectively controls established arthritis and improves mechanical allodynia. However, its therapeutic potential is significantly enhanced when utilized after Dex‐induced suppression of inflammation, reducing cumulative disease burden and providing more robust pain modification.

## 3 DISCUSSION

### 3.1 Mechanical allodynia in SKG mice

This study characterized mechanical allodynia associated with arthritis progression in SKG mice and demonstrated the utility of the model for investigating arthritis‐associated pain and potential interventions. Male and female SKG mice show significant mechanical allodynia without changes in thermal sensitivity, suggesting arthritis‐driven mechanosensory dysfunction rather than alterations in thermal nociception, and a role of mechanoreceptor‐initiated nociceptor traffic.^22^, ^23^, ^24^ The lack of changes in thermal nociceptive thresholds aligns with prior work with the K/BxN and CAIA arthritis models.^22^, ^23^, ^24^ These observations, while common, are nonetheless surprising given the cyclooxygenase‐sensitive inflammatory phenotype which normally serves to sensitize thermal transducing poly modal nociceptors.^40^ While arthritis progressed consistently in males and females, more severe disease was associated with females as assessed by higher clinical scores. Both sexes developed significant mechanical allodynia by days 5–6, which correlated with disease severity. As no prior studies have characterized pain behavior in SKG mice, our work establishes this model as a foundational platform for studying arthritis‐associated pain and its therapeutic modulation.

### 3.2 Analgesic profile of the SKG arthritis

The analgesic pharmacology in SKG mice depended on the stage of disease. In established arthritis, both ibuprofen and gabapentin alleviated mechanical allodynia; however, during the chronic phase, only gabapentin remained effective. This shift is consistent with prior work which reflects a transition from inflammation‐driven pain, which is responsive to cyclooxygenase inhibition produced by an NSAID, to a neuropathic‐like state requiring gabapentinoids.^25^ However, the anti‐inflammatory efficacy of ibuprofen in this model, as well as the potential contribution of joint and bone structural damage to pain at later stages, cannot be ruled out. Further studies with shorter disease duration and additional assessment of tissue pathology could shed more light on these factors. Similar transitions have been observed in other arthritis models, where chronic pain reflects changes in afferent evoked neuraxial processing evolving to a neuropathic phenotype.^25^, ^41^, ^42^ These findings parallel clinical observations where early arthritis pain is primarily inflammation‐driven, while chronic pain involves complex neuroimmune interactions, which we believe underscores the need for stage‐specific therapeutic strategies.^43^, ^44^

### 3.3 Pain modification with ATRA‐PLGA MP and CTLA‐4‐Ig

We developed a 50 μm ATRA‐PLGA MP for intra‐articular drug delivery. This microparticle size was chosen to ensure prolonged joint retention and sustained controlled release of ATRA while minimizing systemic exposure.^45^ The larger size maintains localized ATRA concentrations in the joint at therapeutically relevant levels to modulate immune responses.^46^ In vitro studies showed that the release profile was strongly influenced by albumin concentration, with direct proportionality between the albumin concentration and the fraction released in the early phase. These observations suggest that albumin likely enhances ATRA solubility.^47^ However, in simulated synovial fluid containing albumin, hyaluronic acid, mucoprotein, and urea, among other components, the release rate of ATRA was greatly reduced. It is likely that the diffusion of ATRA was influenced by the high viscosity and components of the artificial synovial fluid.

ATRA‐PLGA MP monotherapy prolonged therapeutic effect in the early stages (day 3) of arthritis, effectively alleviating disease severity, and maintaining lower clinical scores even after a single IA injection albeit with limited effect on mechanical allodynia. Our previous work identified increased CTLA‐4 expression in joint‐localized dendritic cells and CD4^+^ T cells following nanoparticle treatment, which was associated with reduced arthritis severity and protection against bone and cartilage damage.^37^ Results from the “Abatacept Reversing subclinical Inflammation as measured by MRI in ACPA positive Arthralgia” (ARIAA) trial showed that CTLA‐4 Ig treatment delayed disease onset for up to 18 months in and the “Arthritis Prevention In the Pre‐clinical Phase of Rheumatoid Arthritis with Abatacept” (APIPPRA) trial showed a delay for up to 24 months.^34^, ^36^ Given these findings, we included CTLA‐4‐Ig as a comparator and observed that ATRA‐PLGA MP potentiated its therapeutic effects, particularly in reducing mechanical allodynia. While CTLA‐4‐Ig operates via generalized immunosuppression, our data suggest that co‐administration with ATRA‐PLGA MP may enhance both early disease control and pain modulation.

In established arthritis, Dex was effective in rapidly controlling inflammation and providing a pain alleviating effect. However, the effect was transient, with disease severity rebounding soon after the discontinuation of Dex injections. This result aligns with previous findings showing that while corticosteroids provide moderate short‐term relief from pain and inflammation, their long‐term efficacy remains limited.^38^ Combining ATRA‐PLGA MP with Dex extended therapeutic benefits by modulating cumulative disease burden and improving mechanical allodynia.

### 3.4 Sexual dimorphism

Sexual dimorphism in SKG arthritis modeled the known sex differences in rheumatoid arthritis. Female SKG mice exhibited more severe arthritis, requiring a higher Dex dose for effective suppression. Strikingly, a correlation analysis showed that arthritis in female mice was associated with lower mechanical sensitivity and associated pain behaviors. This observation aligns with previous studies suggesting that female mice may engage compensatory mechanisms, such as neuroimmune modulation, that reduce pain sensitivity despite increased inflammation.^48^ The sexual dimorphism observed in SKG mice suggests the possibility for modeling personalized therapeutic approaches that account for variations in disease severity and pain processing between sexes.

### 3.5 Limitations of the study

While our studies have focused on arthritis‐associated pain and potential treatment strategies in the SKG model of RA, further development would likely benefit from additional studies, some of which are discussed here. Chronic arthritis pain is multifactorial, often persisting despite resolution of inflammation, and may involve neuropathic, central sensitization, and structural components. Capturing chronic pain states poses inherent challenges in preclinical models due to the complexity of its etiology and the limitations of current behavioral assays. Although mechanical allodynia was comprehensively characterized in this study, other pain modalities such as spontaneous pain, thermal hypersensitivity beyond heat, and functional impairment could provide additional insight into the pain phenotype. Additional assessments focused on the mechanisms underlying mechanisms driving sexual dimorphism could further our understanding in this regard. The role of joint and bone structural damage in mediating pain, especially during chronic disease, warrants further investigation using histopathological and imaging approaches. Studies with shorter and more finely resolved disease timelines, as well as broader drug dosing windows, could better define the temporal dynamics of pain mechanisms and therapeutic response. While our study demonstrates that intra‐articular ATRA‐PLGA microparticles, alone or in combination with systemic immunosuppressants, can provide pain alleviation in the SKG arthritis model, an assessment of the durability of the effect will determine the feasibility of extended pain alleviation or even disease remission.

## 4 CONCLUSIONS

Overall, our findings establish SKG mice as a robust model for arthritis‐induced mechanical allodynia, emphasizing the need for stage‐specific treatment strategies. ATRA‐PLGA MP demonstrated therapeutic potential, both as a monotherapy and in combination with immunomodulatory agents or corticosteroids, for improving arthritis management and patient‐tailored therapies for rheumatoid arthritis.

## 5 MATERIALS AND METHODS

### 5.1 Study design

The study aimed to assess pain responses and therapeutic efficacy in SKG mice, a T cell‐driven autoimmune arthritis model resembling RA, during disease progression. Therapeutic interventions involved the intra‐articular injection of ATRA‐PLGA MP into a single joint (ankle, unilateral), either at an early stage (day 3) or established in arthritis progression (day 14). These treatments are compared to existing therapy options or used in combination with them. To examine potential sexual dimorphism in arthritis progression and treatment response, the same studies were conducted in male and female SKG mice (8–12 weeks old). Arthritis was induced by a single intraperitoneal injection of mannan, and disease progression was monitored through clinical arthritis scoring conducted at least twice weekly.^15^ Pain sensitivity was assessed through mechanical allodynia using the von Frey filament test and thermal latency by the Hargreaves method.^20^, ^49^

All experimental procedures were approved under protocol S17160 by the Institutional Animal Care and Use Committee (IACUC) of the University of California, San Diego, USA, and adhered to ethical research guidelines.

Assessed outcomes include clinical arthritis scores and PWT alterations for pain sensitivity. Temporal disease progression and treatment efficacy were analyzed using area under the curve (AUC) metrics derived from clinical scoring and mechanical allodynia testing. AUC was calculated for each individual mouse using the trapezoidal rule based on standard protocols.^49^ The baseline (*Y* = 0) was defined depending on the context of each experiment to ensure consistent and meaningful comparisons across treatment groups. Specifically, for the temporal profiling of pain behavior in SKG mice after mannan‐induced arthritis, the baseline was set to 0 for clinical score, thermal latency, and PWT, as all animals began without clinical symptoms. For the evaluation of therapeutic efficacy at the early stage of arthritis, the baseline for clinical score was set to 0 (as treatment began on day 0 when all mice had no clinical signs), and the baseline for PWT was set individually based on the pre‐treatment threshold for each mouse. For the assessment of therapeutic effectiveness at the established phase of arthritis, interventions began on day 14, and therefore, baseline values for clinical score and PWT were set to the corresponding day 14 values for each mouse. This approach was used to evaluate how well each treatment suppressed arthritis progression and pain: lower AUC values for clinical scores indicate milder disease progression, whereas lower AUC values for PWT reflect greater mechanical allodynia (i.e., worse pain sensitivity). Clinical score AUC is presented in clinical score × days, and PWT AUC in grams × days. Units of the AUC values are in the figure captions. Clinical score AUC is presented in score × days, and PWT AUC in grams × days. Linear regression analysis was applied to assess mechanical and thermal allodynia in the temporal profiling of pain behavior in SKG mice after mannan‐induced arthritis. Statistical comparisons were performed using two‐tailed paired or unpaired t‐tests for normally distributed data, as well as one‐way and two‐way ANOVA for multifactorial analyses. A significance threshold of *p* < 0.05 was applied. Correlation analysis was conducted using data from male and female experimental groups to assess the relationship between pain sensitivity and clinical scores throughout arthritis progression. Data collected from day 0 (arthritis induction with mannan) to day 13 (pre‐treatment) were included in the analysis. Pearson's correlation coefficient (*r*) was calculated to quantify the strength and direction of the association between PWT and clinical scores. To compare the slopes of the linear regression lines, the average slope and standard error were calculated, and an *F*‐test was performed. The number of data pairs (*n*) used in the correlation analysis was considered as the sample size for statistical comparison of slopes. Data are presented as mean ± standard error, and statistical significance was set at *p* < 0.05. Data analysis was conducted using GraphPad Prism version 10.1.2. Animal studies were not blinded.

### 5.2 Materials

Poly (lactic‐co‐glycolic acid) (PLGA, 75:25, AP018, lot: 201204RAI‐C, M_w_ 45–55 kDa) was acquired from Akina. All‐trans retinoic acid (ATRA, BML‐GR100‐0500, lot: 06011830) was obtained from Enzo. Dichloromethane (DCM, D143‐1, lot: 194105) and dimethyl sulfoxide (DMSO, D128‐500, lot: 194474) were purchased from Fisher Chemical. Mannan (M7504‐5G, lot: SLCF4977) and polyvinyl alcohol (PVA, 363146‐500G, lot: MKCF9787, M_w_ 85–124 kDa) were procured from Sigma‐Aldrich. Recombinant human IgG1 Fc (BE0096, lot: 829223N1) and CTLA‐4‐Ig (BE0099, lot: 826622J1) were purchased from Bio X Cell. Ibuprofen (IB100, lot: 4NA0006) and gabapentin (G1092, lot: 4KH0004) were acquired from Spectrum Chemical. Bovine serum albumin (BSA, 700‐100P, lot: C121024) was purchased from GeminiBio. Artificial synovial fluid was purchased from Biochemazone and customized to pH 7.0 (BZ183, lot: BZ183‐0625A). Dexamethasone (Dex, V1 501012, lot: 240659) was acquired from Vet One. The Semmes‐Weinstein monofilaments Touch Test™ Sensory Probes (Catalog #95060‐230) were used in this study and purchased from Stoelting (Supplier Number: 58011).

### 5.3 Microparticle synthesis

Blank‐PLGA MP and ATRA‐PLGA MP were prepared using an oil‐in‐water emulsion method, based on a modified procedure from previous studies.^17^ In brief, 1 g of PLGA was dissolved in 6.67 mL of DCM. For ATRA‐PLGA MP, 20 mg of ATRA was added to the PLGA solution in DCM and mixed thoroughly until fully dissolved. This solution was then gradually added dropwise into 200 mL of a 0.3% (w/v) PVA solution under continuous homogenization for 2 min at 2000 rpm using a Silverson™ homogenizer to form the emulsion. The resulting emulsion was stirred at 300 rpm for 4 h at room temperature while shielded from light. To remove agglomerates and ensure uniformity, the suspension was vacuum‐filtered sequentially through 100 and 30‐μm filters. Particles collected on the 30‐μm filter were washed four times with sterile deionized water, then frozen and lyophilized and stored at −20°C. The size and morphology of the microparticles were subsequently characterized using SEM.

### 5.4 Scanning electron microscopy

Lyophilized microparticles were gently agitated to produce a free‐flowing powder. A small quantity of the powder was deposited onto carbon adhesive tape secured to an SEM stub. Any excess material was removed by carefully blowing compressed air across the stub's surface. The prepared stubs were then sputter‐coated using an Emitech K575X Sputter Coater under an argon atmosphere at a pressure of 4 × 10^−3^ mbar. A thin iridium layer was applied using an 85‐mA current for 20 s to enhance conductivity and image quality. Once coated, the stubs were placed in a scanning electron microscope (FEI Quanta FEG 250) and imaged at an accelerating voltage of 5 kV in a high‐vacuum mode. Particle size was determined by analyzing the SEM micrographs using ImageJ software. Measurements were performed on well‐defined particles from the images, ensuring accurate size distribution calculations.

### 5.5 Zeta potential easurements

The zeta potential of Blank‐PLGA MP and ATRA‐PLGA MP was measured using Zetasizer (Malvern Instruments). To prepare samples, 5 mg of particles were suspended in 10 mL of deionized water and sonicated and vortexed briefly to ensure uniform dispersion. The suspension was then transferred to a disposable folded capillary cell, ensuring no air bubbles were present. Measurements were carried out at room temperature (25°C) with the instrument set to the default dielectric constant and refractive index values for water. Each sample was measured in triplicate, and the zeta potential distribution graphs were reported.

### 5.6 Protein corona formation on macroparticles

The formation of a protein corona on Blank‐PLGA MP was assessed using BSA as a model protein. Briefly, 5–10 mg of Blank‐PLGA MP were suspended in 1 mL of BSA solution (10 mg × mL) in Eppendorf tubes. The tubes were incubated on a shaker at 37°C for 2 h to allow protein adsorption. Following incubation, the particles were centrifuged (1000 g for 5 min) and washed five times with 1 x PBS to remove unbound protein. To confirm protein binding to the microparticles, a bicinchoninic acid (BCA) assay was performed on the particles exposed to BSA, with the BSA solution used as a positive control and Blank‐PLGA MP incubated with 1 x PBS as a negative control, following the manufacturer's instructions. Absorbance values were recorded in triplicate. Due to ATRA's interference with the reagents in the BCA assay, the analysis was conducted exclusively on Blank‐PLGA MP.

### 5.7 Loading capacity of ATRA‐PLGA MP

To determine the loading capacity of ATRA‐PLGA MP, 10 mg of the microparticles were dissolved in 10 mL of DMSO under continuous shaking until complete dissolution was achieved. The absorbance of the resulting solution was measured using a Nanodrop UV–vis spectrophotometer, and the concentration of ATRA was calculated based on a standard curve. The ATRA concentration (mg × mL^−1^) was converted into the total amount of ATRA (mg), and the loading capacity was calculated relative to the initial weight of the particles using the following formula:

$$\text{Loading Capacity}\;\left(\%\right)=\frac{\text{Mass of ATRA loaded}}{\text{Mass of PLGA}\;\mathrm{MP}}\times 100\%$$

### 5.8 In vitro ATRA release assay

To assess the release profile of ATRA from ATRA‐PLGA MP, the particles were suspended at a concentration of 10 mg × mL^−1^ in PBS containing 0.1, 10, or 50 mg × mL^−1^ BSA, or in simulated synovial fluid. The suspension was incubated at 37°C with intermittent shaking to simulate physiological conditions. At each time point, the microparticles were centrifuged at 1000 g for 5 min to separate the supernatant, which was carefully collected without disturbing the particle pellet. The supernatant was replaced with fresh release buffer, and the particle pellet was resuspended by vigorous vortexing. The collected supernatant was frozen, lyophilized, and subsequently reconstituted in DMSO. The concentration of ATRA was then determined using a Nanodrop UV–vis spectrophotometer and a standard curve.

### 5.9 SKG arthritis model

Adult male and female SKG mice were used for this study. They were housed in isolated ventilated racks in a 12:12 h light or dark cycle with controlled humidity and temperature (22°C). Food and water were available ad libitum. Arthritis onset was induced in 8–12‐week‐old mice via intraperitoneal injection of 20 mg of mannan dissolved in 200 μL of sterile 1 x PBS, following established protocols.^17^ Disease progression was monitored twice weekly using a standardized clinical scoring system. Each forepaw and hindpaw was scored individually based on visible swelling: 0 for no swelling, 0.5 for mild to moderate swelling, and 1.0 for severe swelling, with an additional 0.1 assigned for each swollen digit.^17^ The clinical score for each mouse represented the aggregate score across all paws, with a maximum possible score of 5.8. Mice reaching a clinical endpoint score of 5.5 were euthanized according to IACUC guidelines. Mice that failed to develop arthritis after mannan injection or showed symptoms before injection were excluded, with misinjection rates in rodents typically ranging from 10% to 25%. Mice were randomly assigned to experimental groups using stratified randomization to ensure comparable disease severity between groups at baseline. In the gabapentin and ibuprofen analgesic studies, randomization was based on baseline PWT measurements to ensure comparability in pain sensitivity between groups prior to treatment. In other studies, randomization was based on clinical arthritis scores, such that the average disease severity was similar between groups at the time of treatment initiation.

To assess the temporal profiling of pain behavior in SKG mice after mannan‐induced arthritis, both male and female SKG mice were injected with mannan IP on day 0. The development of arthritis was monitored through clinical scoring, while pain behavior was evaluated using the von Frey and Hargreaves methods, with the study concluding 13 days after the mannan injection.

To evaluate the therapeutic efficacy of gabapentin and ibuprofen, arthritis was induced in SKG mice via IP injection of mannan as described above. On days 15 and 46 post‐induction, mice received an IP injection of either gabapentin (100 mg × kg^−1^) or ibuprofen (100 mg × kg^−1^), while the sham group received a vehicle injection (10% DMSO/10% Tween‐80 in PBS) to evaluate analgesic effects. Mechanical allodynia thresholds were assessed 1 day prior to treatment and 1 h post‐treatment in the control and treated groups.

To determine whether ATRA‐PLGA MP injections are associated with pain, naïve SKG mice received a single IA injection into one hind ankle and had the mechanical allodynia measured by von Frey test in the ipsilateral (injected) and contralateral (non‐injected) paw on days 1, 4, and 7 post‐injection.

To evaluate the therapeutic efficacy of ATRA‐PLGA MP during the early stage of arthritis, arthritis was induced with mannan as described above. To benchmark ATRA‐PLGA MP against a standard‐of‐care treatment, CTLA4‐IgG was administered IP at a dose of 10 mg × kg^−1^ daily from days 0 to 2. IgG‐treated (10 mg × kg^−1^ daily from days 0 to 2) and vehicle‐treated (1 × PBS) mice served as the negative controls. On day 3 of the post‐mannan injection, mice received a single IA injection into the hind ankle joint. Treatment groups included 20 μg of ATRA‐PLGA MP suspended in 20 μL of sterile PBS, or a sham injection of Blank‐PLGA MP. Clinical scores and mechanical allodynia were assessed for 7 days following IA treatment, with the study concluding 10 days after the mannan injection.

To assess the therapeutic effectiveness of ATRA‐PLGA MP at the established phase of arthritis, mannan was used to induce arthritis as described above. From days 14 to 16 post‐induction, male mice received daily IP injections of dexamethasone at a dose of 1 mg × kg^−1^, and female mice received 5 mg × kg^−1^, or saline as a negative control. On day 17, mice were administered either ATRA‐PLGA MP or Blank‐PLGA MP via IA injection as described above. Clinical scores and mechanical allodynia were tracked bi‐weekly for an additional 10 days post‐treatment, with an endpoint at 27 days after mannan injection.

### 5.10 Mechanical allodynia

Mice were placed in individual compartments measuring 3 × 3 × 6 inches (*W*, *L*, *H*) within a box that allowed free movement. The floor of each compartment was constructed of wire mesh, providing access to the plantar surface of the hind paw. Prior to testing, all mice were acclimated to the testing environment for at least 2 h to minimize stress. Testing commenced only when the mice appeared calm, with no signs of stressed behavior or excessive movement.

Mechanical sensitivity was assessed by von Frey filament using the up‐down method as previously described.^50^ Filaments with different diameters and stiffness (pressure from 0.02 to 2 g) were applied to the center of mice's hind paws until eliciting paw withdrawal.^50^ This method involved sequential application of filaments in an ascending or descending order until a response reversal was observed. Following the first reversal, the up‐down sequence was repeated for several cycles, collecting a total of six responses. The withdrawal threshold was calculated using standard tables. Mechanical values for the left and right hind paws were measured and averaged to produce a single data point per measurement day.

### 5.11 Thermal latency

Thermal withdrawal latency was measured using a modified Hargreaves apparatus equipped with a glass platform maintained around 35–40°C (Department of Anesthesiology, University of California San Diego). Mice were individually placed in plexiglas cubicles on the glass surface and allowed to acclimate for 30 min before testing. A focused thermal stimulus, generated by a projection bulb positioned beneath the glass platform, was applied to the mid‐plantar region of the hind paw. Withdrawal latency was defined as the time taken for the paw to exhibit a brisk withdrawal response, which was detected by photodiode motion sensors that stopped the timer and ended the stimulus. If no withdrawal response occurred within 20 s, the stimulus was automatically terminated to prevent tissue damage (cut‐off time). Thermal latency was calculated as the average of three measurements taken from each hind paw.

## AUTHOR CONTRIBUTIONS

**Maksim Dolmat**: Conceptualization, methodology, validation, formal analysis, investigation, resources, data curation, writing—original draft, writing—review and editing, and visualization. **Julia Borges Paes Lemes**: Investigation, writing—review and editing. **Wade T. Johnson**: Investigation. **Elizabeth L. Wilkinson**: Investigation. **Tony L. Yaksh**: Conceptualization, resources, methodology, supervision, writing—original draft, writing—review and editing. **Nunzio Bottini**: Conceptualization, methodology, supervision, writing—original draft, writing—review and editing. **Nisarg J. Shah**: Conceptualization, funding acquisition, project administration, resources, supervision, writing—original draft, writing—review and editing.

## CONFLICT OF INTEREST STATEMENT

N.J.S. and N.B. are academic founders of Tekhona Inc. and have an equity interest in the company. The terms of this arrangement have been reviewed and approved by their institutions in accordance with conflict of interest policies. The remaining authors declare no relevant conflicts of interest.

## Supporting information


**Data S1:** Supplementary Figure

## Data Availability

The data that support the findings of this study are available from the corresponding author upon reasonable request.
