# Supplementary material for: Disease modifying biomaterials for modulating mechanical allodynia in a preclinical model of rheumatoid arthritis
Source: Bioeng Transl Med. 2025 Jul 31;10(6):e70054. doi: 10.1002/btm2.70054 (PMC12617560; doi:10.1002/btm2.70054)
Supplement: Supplementary file 1 — Data S1: Supplementary Figure [file BTM2-10-e70054-s001.pdf]

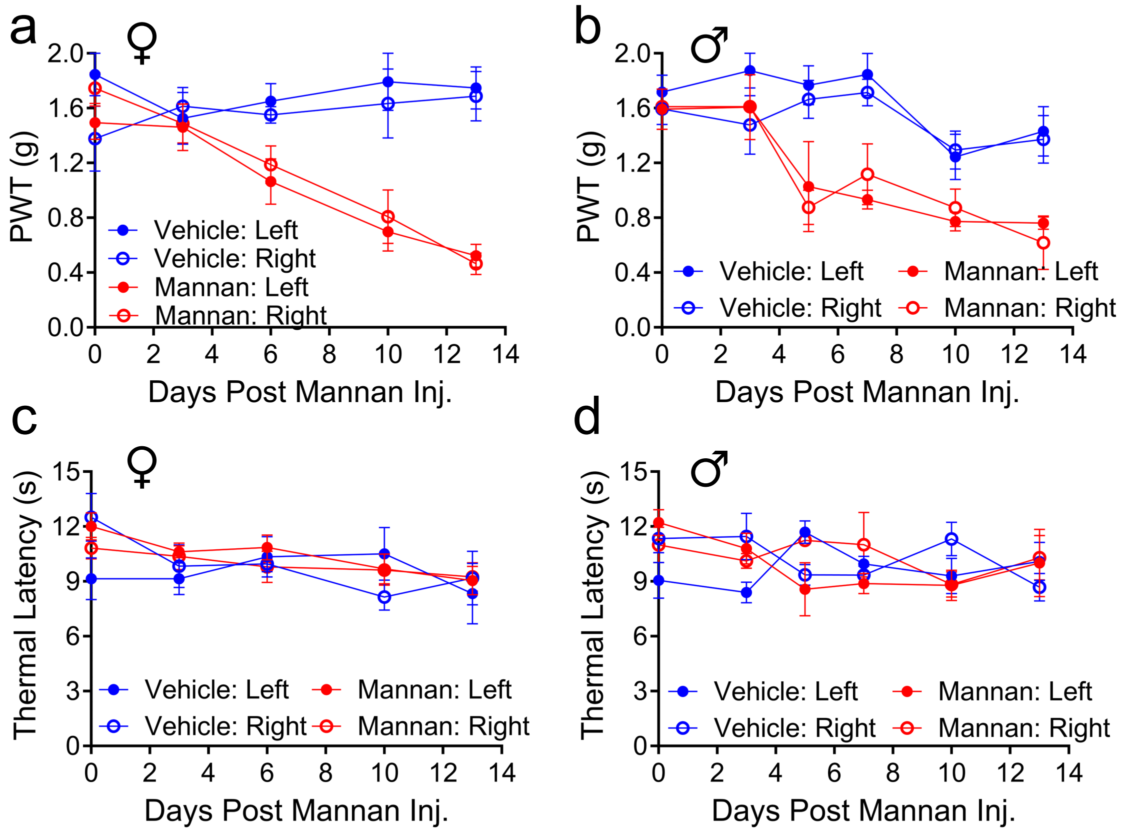

Figure S1. SKG mice were injected intraperitoneally with mannan (red) or vehicle (1x PBS; blue) on day 0, and pain behavior was assessed through von Frey mechanical sensitivity testing (a, b) and thermal paw withdrawal latency using the Hargreaves method (c, d). Paw withdrawal thresholds (PWT) in response to mechanical stimulation and thermal withdrawal latency were measured in both female (a, c) and male (b, d) mice, with no differences observed between the left and right paws. Data are presented as mean ± SEM.

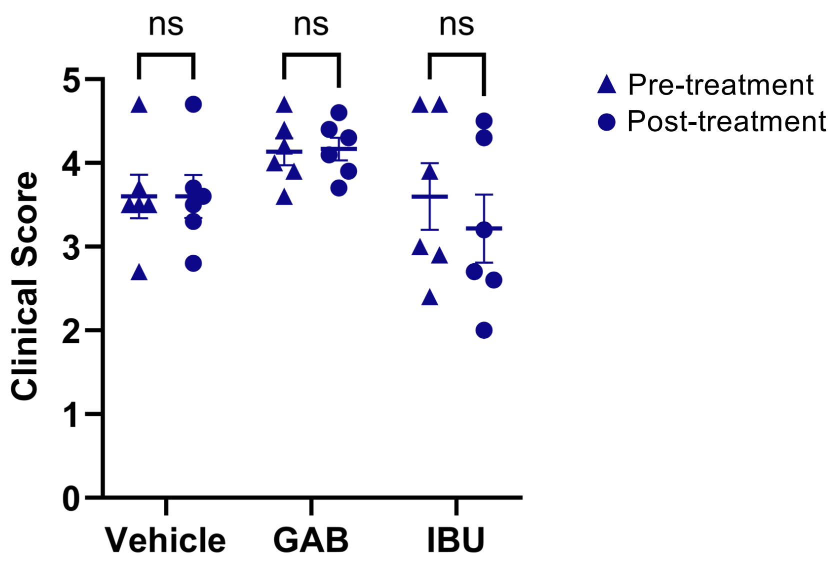

Figure S2. Clinical scores of SKG mice at the established stage of arthritis (day 46), measured before and after intraperitoneal administration of vehicle, gabapentin (GAB, 100 mg × kg-1) or ibuprofen (IBU, 100 mg × kg-1). Two-way ANOVA demonstrated no significant differences between pre-treatment and post-treatment across all treatment groups.

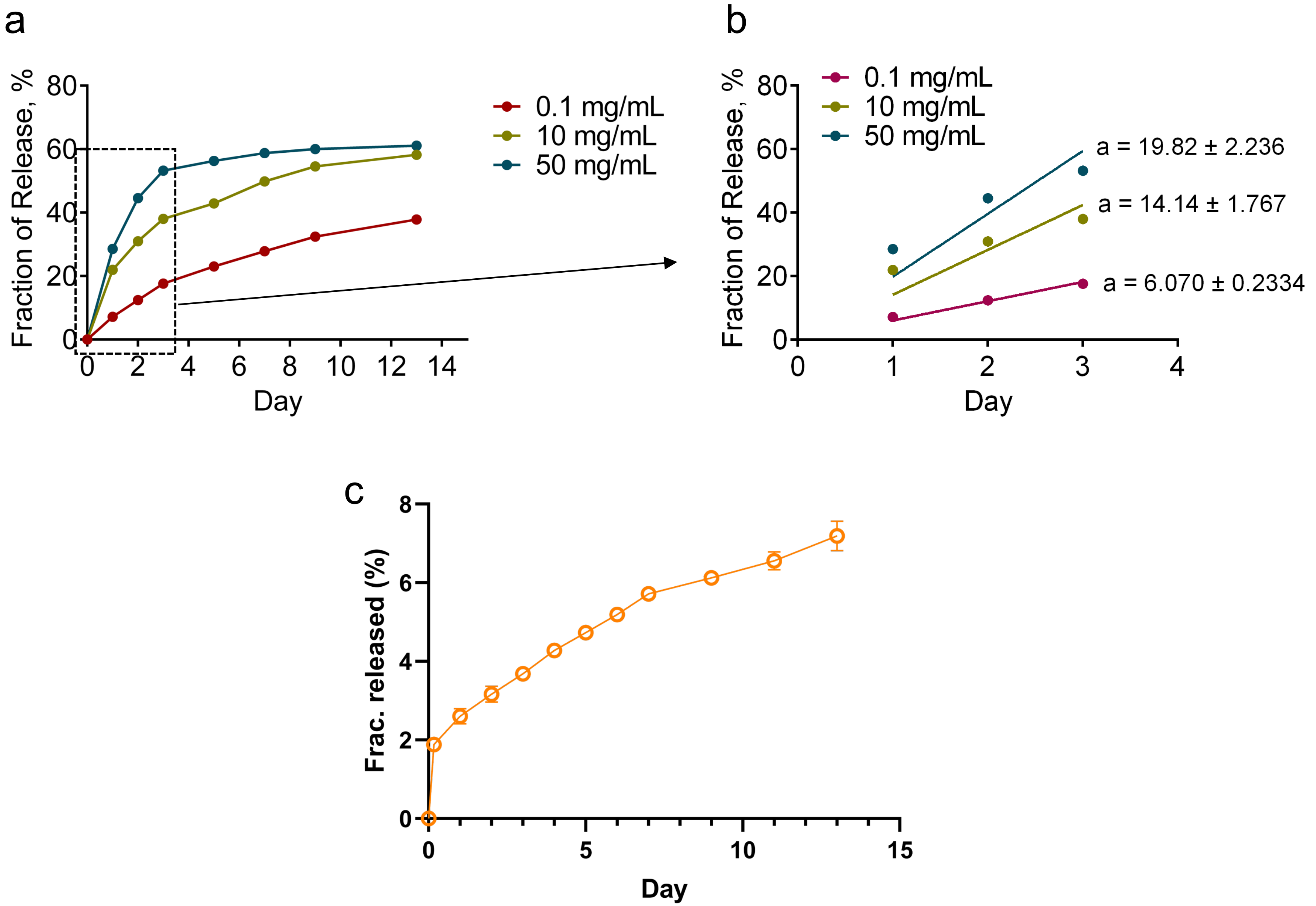

Figure S3. (a) The cumulative fraction of ATRA released over 14 days was measured in the presence of 0.1 mg/mL (dark red), 10 mg/mL (dark yellow), and 50 mg/mL (dark teal) BSA, with higher protein concentrations accelerating release. (b) A magnified view of the first three days shows a near-linear release profile, with release rates calculated for each condition. The 10 mg/mL BSA condition was chosen to approximate albumin concentration in synovial fluid, while 50 mg/mL represents serum albumin levels. (c) The cumulative fraction of ATRA released in artificial synovial fluid over a 14-day period.

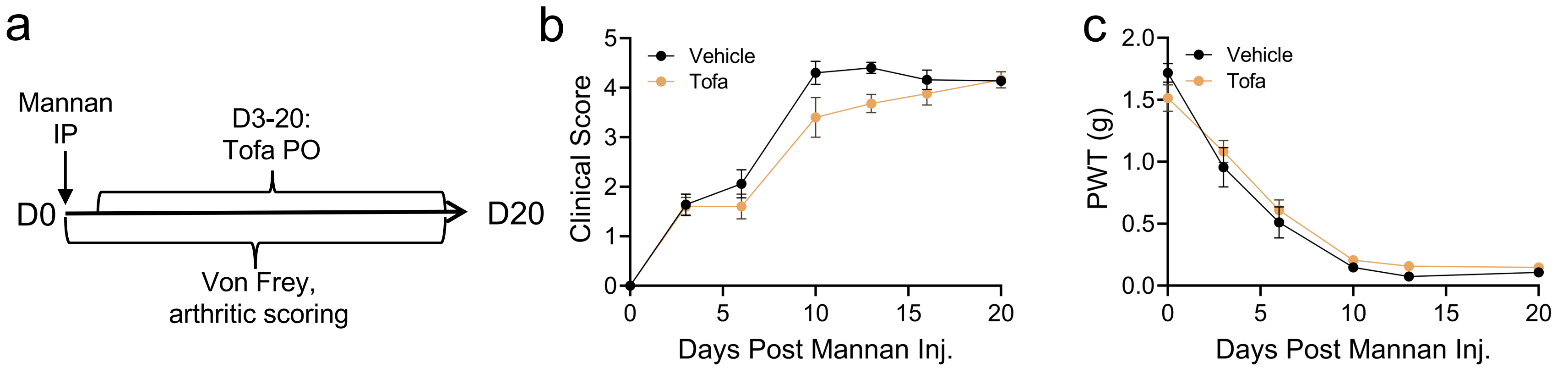

Figure S4. (a) Schematic representation of the experimental timeline: female SKG mice were injected intraperitoneally with mannan on day 0 to induce arthritis. Starting on day 3, mice were treated daily with either tofacitinib (Tofa) or vehicle (1x PBS) until the study endpoint (day 20). (b) Clinical arthritis scores were assessed over time, showing disease progression in both treatment groups. (c) Pain sensitivity was measured using von Frey testing, with paw withdrawal threshold (PWT) decreasing similarly in both groups. Data are presented as mean ± SEM.

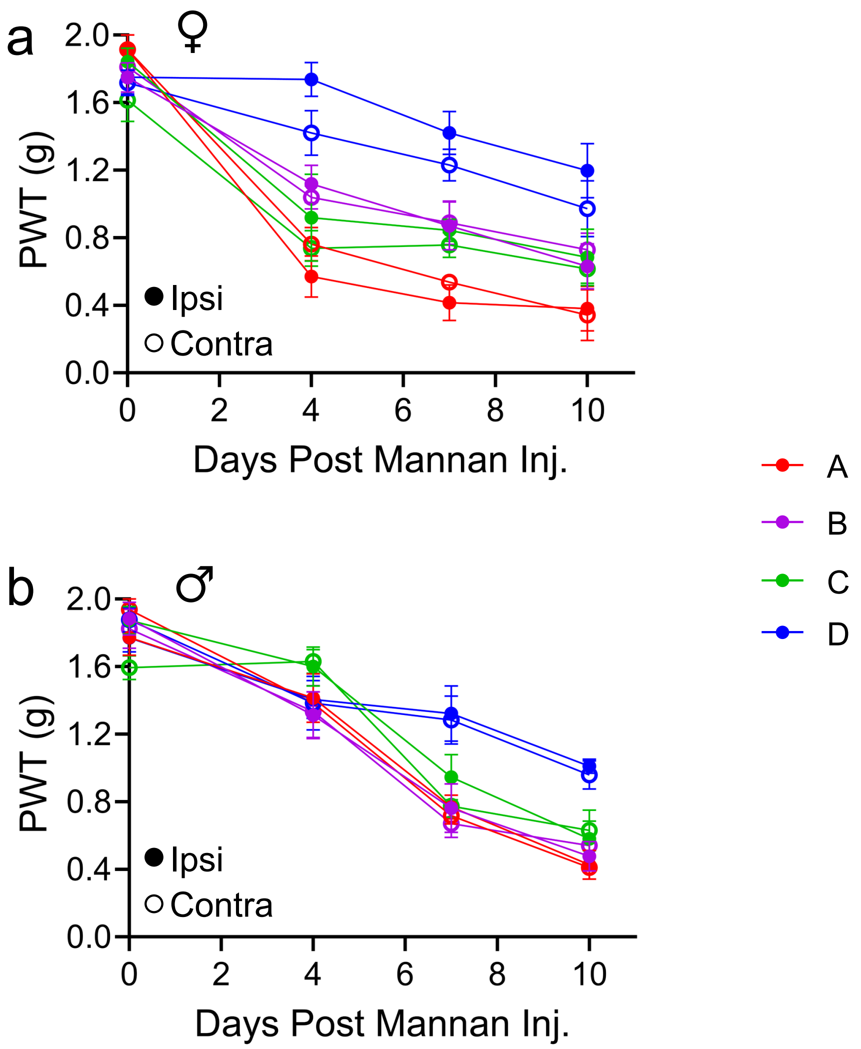

Figure S5. Mechanical allodynia in female (a) and male (b) mice, assessed via von Frey testing at the ipsi and contra ankles.

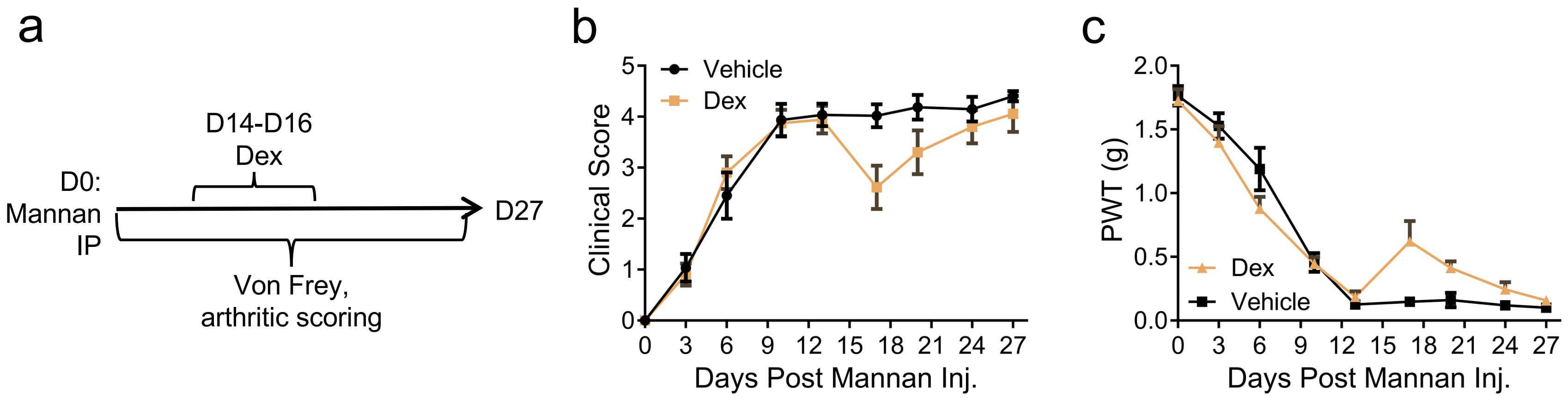

Figure S6. (a) Experimental timeline: Female SKG mice were injected intraperitoneally with mannan on day 0 to induce arthritis. From days 14 to 16, mice were treated intraperitoneally with either dexamethasone (Dex, 1 mg/kg) or vehicle (4.75% ethanol in PBS). (b) Clinical arthritis scores were monitored over time, showing a temporary reduction in disease severity following Dex treatment. (c) Pain sensitivity was assessed using von Frey testing, with paw withdrawal threshold (PWT) decreasing in both groups over time, showing a small transient recovery after Dex treatment. Data are presented as mean ± SEM.
